# Experimental validation of RNA interference technologies for improved control of barber’s pole worm

**DOI:** 10.1186/s13567-025-01633-6

**Published:** 2025-10-14

**Authors:** Jingju Zhang, Fei Wu, Zhendong Du, Jiaming Yu, Haibei Lin, Shengjun Jiang, Hongning Tang, Danru Bu, Xueqiu Chen, Yi Yang, Aifang Du, Guangxu Ma

**Affiliations:** 1 Institute of Preventive Veterinary Medicine, College of Animal Sciences, Zhejiang University, Hangzhou, 310058 China; 2 ZJU-Xinchang Joint Innovation Centre (TianMu Laboratory), Gaochuang Hi-Tech Park, Xinchang, 312500 China; 3 College of Veterinary Medicine, Anhui Agricultural University, Hefei, 230036 China

**Keywords:** *Haemonchus contortus*, adaptation to parasitism, RNA interference, nematode control

## Abstract

Over the past decade, small RNA technologies have been proposed for improved control of parasitic worms. Although achievements have been made in the identification of target candidates and in the improvement of reverse genetic tools, few of those have been tested in domestic animals. In this work, crucial genes (i.e., *daf-9/cyp-22a1*, *bli-5* and *HCON_00083600*) involved in the developmental transition (i.e., activation, moulting and haem utilisation) from the infective L3 stage to the parasitic L4 stage of *Haemonchus contortus* (the barber’s pole worm commonly found in small ruminants) in vitro were identified and verified using RNA interference (RNAi) technologies during the adaptation to parasitism of this parasite in vivo. Silencing each of the *daf-9/cyp-22a1*, *bli-5* and *HCON_00083600* genes in the infective larvae of *H. contortus* resulted in compromised larval development and viability in vitro, and silencing of either the *daf-9/cyp-22a1*, *bli-5* or *HCON_00083600* gene led to a marked reduction in the faecal egg count and worm burden in sheep. In conclusion, the results demonstrate that genes involved in larval activation, moulting and haem utilisation of *H. contortus* are target candidates, and the application of RNAi technologies for better control of these and related parasitic nematodes is promising, preferably with an improved RNAi tool for efficient and long-lasting effects in host animals.

**Supplementary Information:**

The online version contains supplementary material available at 10.1186/s13567-025-01633-6.

## Introduction

*Haemonchus contortus*, also known as the barber’s pole worm, is a haematophagous (blood-feeding) nematode parasite that primarily infects small ruminants such as sheep and goats. It causes significant morbidity through anaemia, oedema, weight loss, and, in severe cases, death (referred to as haemonchosis), representing one of the most pathogenic parasitic worms of livestock animals worldwide (cf. [1]). Barbervax, the first commercially available vaccine incorporating the natural intestinal antigens H-gal-GP and H11 has demonstrated efficacy in protecting pregnant and lactating ewes as well as lambs against *H. contortus* infection. However, its vaccination protocol, which requires both primary and booster doses, poses logistical challenges in terms of administration and diminishes its effectiveness when simplified under real-world production conditions [2–4]. Thus, the control of *H. contortus* infection relies heavily on the administration of anthelmintic drugs, such as albendazole, ivermectin, levamisole and its derivatives [5–7]. However, the emergence of drug resistance and multidrug resistance poses inevitable challenges to the control of *H. contortus* infection and the treatment of haemonchosis [8–10]. A deeper understanding of the biological processes of this parasite is needed to identify potential molecular targets and facilitate more effective control strategies against *H. contortus* [11, 12].

The life cycle of *H. contortus* consists of a free-living phase (i.e., the egg, first, second, and third larval stages) in the environment and a blood-feeding phase (i.e., the fourth-larval and adult stages) in host animals [13]. The first-stage larvae (L1s) hatch from eggs, feed on bacteria, moult and develop into the second-stage larvae (L2s). These then moult and become the third-stage larvae (L3s); the infective L3s are ensheathed, developmentally paused, resistant and viable on pasture for months until they are ingested by host animals. In host animals, the L3s are activated, exsheath, moult, and become the fourth-stage larvae (L4s) in the abomasa, which feed on blood, moult, develop to female and male adults, and produce eggs into faeces. Biological processes involved in the developmental transition from the free-living stage to the parasitic stage of *H. contortus* (e.g., larval diapause and activation, moulting, and blood-feeding) have been extensively studied (reviewed in [12, 14]), and genes that are crucial for the survival and development of this parasite have been identified. For instance, the FAU ubiquitin-like and ribosomal protein S30 fusion protein-encoding gene *fau* is involved in larval diapause [15], the ubiquitin- and glutathione S-transferase-encoding genes are important for desiccation tolerance [16], the nuclear hormone receptor-encoding gene *daf-12* is essential for larval activation [17], the acyl-CoA oxidase-encoding gene *acox-1* is vital for larval development [18], and the haem transporter-encoding genes *hrg-1* and *mrp-3* are required for haem utilisation in the blood-feeding *H. contortus* [19, 20]. The essentiality of these molecules in *H. contortus* has been somewhat tested in animals in terms of nematode infection, development, and reproduction [20–22], leading to the identification of target candidates for the intervention of *H. contortus* infection.

RNA interference (RNAi) is a conserved biological response to double-stranded RNA (e.g., endogenous parasitic or exogenous pathogenic nucleic acids) that mediates posttranscriptional silencing of protein-coding genes. This RNA technology has been extensively used in nematode functional genomics [23, 24] and in target screening and validation for *H. contortus* [25–31]. Although inconsistent susceptibility and variable efficiency of gene silencing have been reported in *H. contortus* and other nematodes [26, 30, 32, 33], the factors affecting the susceptibility and stability of RNAi in this parasite have been investigated and comprehensively discussed [11, 34–36]. The application of RNAi technology to identify essential genes and target candidates for improved control of *H. contortus* has been proposed for a decade [32, 37], but little progress in terms of clinical trials has been made in domestic animals.

In this work, critical genes involved in the key biological processes of *H. contortus* in host animals (i.e., larval development after activation, moulting and blood-feeding) were identified in vitro, and the infective larvae of this parasite with these genes knocked down using RNAi (feeding and soaking methods) were tested in vivo to provide a solid proof of concept for RNAi technologies in the control of barber’s pole worm in small ruminants.

## Materials and methods

### Ethics statement

Helminth-free sheep (three months old) and rabbits were raised under well-controlled conditions. All animal experiments were approved by the Experimental Animals Ethics Committee of Zhejiang University (Permit No. ZJU20241015), Hangzhou, People’s Republic of China. The study complied with all relevant ethical regulations for animal use.

### Genomic datasets

The publicly available genomic datasets of parasitic nematodes of animals (e.g., *Acanthocheilonema viteae*, *Ancylostoma* sp., *Angiostrongylus cantonensis*, *Anisakis simplex*, *Ascaris* sp., *Brugia* sp., *Cylicostephanus goldi*, *Dictyocaulus viviparus*, *Dirofilaria immitis*, *Dracunculus medinensis*, *Elaeophora elaphi*, *Enterobius vermicularis*, *Gongylonema pulchrum*, *Haemonchus* sp., *Heligmosomoides polygyrus*, *Litomosoides sigmodontis*, *Loa loa*, *Necator americanus*, *Nippostrongylus brasiliensis*, *Oesophagostomum dentatum*, *Onchocerca* sp., *Parascaris* sp., *Parastrongyloides trichosuri*, *Pristionchus pacificus*, *Soboliphyme baturini*, *Strongyloides* sp., *Strongylus vulgaris*, *Syphacia muris*, *Teladorsagia circumcincta*, *Thelazia callipaeda*, *Toxocara canis*, *Trichinella* sp., *Trichuris* sp. and *Wuchereria bancrofti*) were accessed from the WormBase ParaSite (version: WBPS15; [38, 39]).

### Gene identification and curation

Genetic information on *cyp* genes in all the nematodes; the moulting-associated genes *bli-3*, *bli-4*, *bli-5*, *phy-2, dpy-18*, *dpy-31, pdi, ppia*, *sec-23*, and *mlt-7*; and *hrg-1* associated with *HCON_00083600* in *H. contortus* were obtained from WormBase (version WBS271; [40, 41]). Gene homologues in parasitic nematodes were inferred on the basis of the Compara database of gene trees at WormBase ParaSite [39, 42]. Multiple sequence analysis was conducted using the Clustal Omega tool [43, 44]. BLAST searches of nucleic acid sequences (e-value < 10^–05^) and InterProScan [45] searches of amino acid sequences of parasitic nematodes against those of *C. elegans* were performed to achieve exhausted gene identification. The identification of gene homologues was manually curated among species, particularly in terms of the phylogenetic aspect of parasitic nematodes (clades I, III, IV and V). Specifically, identified *cyp* genes were curated and integrated with previous identifications [46] and nomenclature with a prefix *cyp* followed by a number for the family, a letter for the subfamily and a number for the specific gene (see [47]), on the basis of their evolutionary conservation and diversification relationships with *cyp* genes in *C. elegans*. Protein structures of the same structural topology were used in protein structure modelling and compared using PyMOL v2.5 (Schrödinger, Inc.).

### Transcriptomic data analysis

Transcriptional analyses of the curated *cyp* and *bli-5* genes were performed during the developmental stages of this parasite by exploiting the transcriptomic datasets publicly available for *H. contortus* [46, 48]. Moreover, transcriptional data for *bli-4* (*WBGene00000254*) and *bli-5* (*WBGene00000255*) of *C. elegans* (*PRJNA13758*) at different developmental stages both inside and outside the egg were analysed and mined from publicly available datasets. In brief, RNA-seq reads from individual developmental stages of the worms were mapped to individual curated coding sequences using Bowtie v.2.1.0 within the software package RSEM v.1.2.11 [49, 50]. Mapped reads were recorded in transcripts per million (TPM). Transcriptional levels of *cyp* and *bli-5* genes among individual developmental stages are displayed in heatmaps generated using pheatmap v.1.0.12.

### Nematode collection and maintenance

The barber’s pole worm *H. contortus* (ZJ strain) was maintained in sheep under well-controlled conditions. Eggs, first-stage (L1s), second-stage (L2s), third-stage (L3s), and fourth-stage (L4s) larvae and adults of this parasitic nematode were collected and maintained using established methods as described previously [17, 18, 20].

### Sheep serum exposure

Exsheathment of the infective L3s of *H. contortus* was conducted in 0.15% v/v sodium hypochlorite (NaClO) at 37 °C for 20 min [17]. The ensheathed L3s (xL3s) of *H. contortus* were cultured (6000 larvae per mL) in Dulbecco’s modified Eagle medium (DMEM; Thermo Fisher Scientific, USA) supplemented with 1 × antibiotic–antimycotic (AA; Gibco) and 10% sheep serum (prepared from helminth-free sheep) in a water-jacketed incubator (Thermo Fisher Scientific, USA) at 38 °C, 10% v/v CO_2_ and 100% humidity. The same volume of DMEM was used as a blank control. After 48 h of culture, the larvae were collected by centrifugation at 600 × *g*, snap-frozen in liquid nitrogen and stored at −80 °C until use.

### Dafadine A treatment

Sterilised xL3s of *H. contortus* (6000 larvae per ml) were suspended in DMEM supplemented with 1 × AA (Gibco) and 100 μM dafadine A (cat. no. SML0736; Sigma‒Aldrich) [51] and incubated at 38 °C with 10% v/v CO_2_ and 100% humidity in a water-jacketed incubator for 7 days. The same volume of DMEM was used as a blank control. After 7 days of culture, the larvae were collected by centrifugation at 600 × *g*, snap-frozen in liquid nitrogen and stored at −80 °C until use.

### RNAi treatment

A feeding method was employed to silence target genes in the free-living L1, L2 and L3 stages of *H. contortus* [18–20]. The target genes in the database were amplified via PCR, cloned and sequenced to obtain high-quality coding sequences from the *H. contortus* ZJ strain. Double-stranded RNA (dsRNA) was designed, synthesised and inserted into L4440 plasmids (Addgene, USA) via *Kpn* I and *Hind* III restriction sites [17]. The recombinant plasmids were subsequently transformed into *Escherichia coli* HT115 competent cells. Eggs of *H. contortus* (*n* = 10 000) isolated from faeces were cultured in 3 mL of culture medium (80% physiological saline, 19% EBSS, 1% yeast extract, 50 µg/mL ampicillin, 2 µg/mL amphotericin B, and 5 µg/mL 5-fluorocytosine) supplemented with *E. coli* HT115 (OD = 0.3) expressing dsRNA at 28 °C with 80% relative humidity for seven days. Larvae fed *E. coli* HT115 bacteria transformed with parental L4440 vector or recombinant L4440 vector expressing dsRNA targeting the *cry1Ac* gene of *Bacillus thuringiensis* (*Bt-cry1Ac*; GenBank accession number GU322939.1, [52]) were used as irrelative controls. The primer sets used for molecular cloning and dsRNA synthesis are provided in Additional file 4.

A soaking method was used to silence the target genes in the xL3 and L4s of *H. contortus* [26]. On the basis of experimentally verified coding sequences of target genes, siRNAs against target genes were designed using siDirect version 2.1 [53, 54], synthesised, mixed with Lipofectamine RNAi MAX reagent (Thermo Fisher Scientific, USA) following the manufacturer’s instructions, and added to the culture medium to achieve a final concentration of 200 pM. The sterilised xL3s were incubated in culture medium in an incubator at 38 °C with 10% v/v CO_2_ for 24, 48, or 72 h. The same volume of medium and siRNAs targeting *Bt-cry1Ac* were used as irrelative controls. The primers used for molecular cloning are provided in Additional file 4.

### Quantitative real-time PCR (qRT‒PCR)

Total RNA was extracted from each sample using TRIzol reagent (Thermo Fisher Scientific, USA) and reversely transcribed into the first strand of cDNA using a ReverTra Ace qPCR RT Kit (Toyobo Co., Ltd., Japan); 50 ng of cDNA was mixed with 10.0 μL of SYBR qPCR master mix (Vazyme, China), 0.4 μL of forward primer, 0.4 μL of reverse primer and 8.2 μL of nuclease-free water as a reaction mixture. The thermocycling program was 95 °C for 30 s, 40 cycles of 95 °C for 10 s and 60 °C for 30 s, and the program for melt curve recording was 95 °C for 15 s, 60 °C for 60 s and 95 °C for 15 s in a CFX96 real-time PCR system (Bio-Rad, Hercules, CA, USA). 18S ribosomal RNA was used as an internal control. Relative transcriptional levels of *daf-9/cyp-22a1 (HCON_00038080)*, *bli-5* (*HCON_00022050*), and *HCON_00083600* genes among developmental stages (i.e., egg, L1, L2, L3, L4 and adult) of *H. contortus* were calculated using the 2^−ΔCt^ method. The transcriptional alterations between treatment conditions (e.g., serum exposure, dafadine A treatment and RNAi) were determined using the 2^−ΔΔCt^ method. At least three technical replicates were included for statistical analysis. The primer sets used for qRT‒PCR are shown in Additional file 4.

### Polyclonal antibody preparation

The coding sequence of *HCON_00083600* was amplified via PCR from cDNA produced using a reverse transcription kit (Toyobo) according to the manufacturer’s protocol. The PCR product was purified, ligated to a linearised pMD19-T vector, amplified in *E. coli*, and then inserted into pET-32a plasmids via the *Bam*H I and *Xho* I restriction sites. The recombinant plasmids were transformed into BL21 bacteria, which were subsequently cultured in medium supplemented with ampicillin and induced with isopropylthio-β-galactoside (IPTG). The recombinant protein was purified using Ni–NTA affinity chromatography and analysed by sodium dodecyl sulphate‒polyacrylamide gel electrophoresis (SDS‒PAGE). The purified recombinant protein was emulsified in Freund’s complete adjuvant and used for the primary immunisation and booster of New Zealand white rabbits. Serum samples were collected from immunised rabbits, subjected to enzyme-linked immunosorbent assay (ELISA), and stored at −80 °C until use.

### Immunofluorescence assay

Adult female and male *H. contortus* were transferred to a filter paper, straightened and fixed in 4% paraformaldehyde solution for 7 days at room temperature. The fixed worms were dehydrated in a graded ethanol series, xylene, embedded in paraffin, sliced into 4 μm sections, and mounted onto slides. After deparaffinization and rehydration, the sections were boiled in antigen retrieval solution, blocked with 10% donkey serum and probed with primary antibody (1:200 dilution) followed by a fluorescent secondary antibody (1:1000 dilution). Nuclei were stained with DAPI (1:1000). The probed slides were mounted with an anti-fade mounting medium and examined under a confocal microscope (Zeiss, Germany).

### Liquid chromatography‒tandem mass spectrometry (LC‒MS‒MS)

Dafadine A-treated and -untreated *H. contortus* larvae were freeze-dried for lipid extraction [17]. Each sample was resuspended in 40% methanol, homogenised, mixed with chloroform (twice the volume of methanol), and centrifuged at 10 000 × *g* for 10 min at room temperature to separate the aqueous and organic phases. The organic phase was retained, dried, and resuspended in methanol for mass spectrometry analysis on an Orbitrap Fusion Lumos mass spectrometer (Thermo Fisher Scientific). The abundance of (25S)-Δ7-dafachronic acid (DA) in treated and untreated samples was determined on the basis of the results of three biological replicates.

### Motility assay

The motility of L1s, L2s, L3s, xL3s, and/or L4s of *H. contortus* (300 larvae in each well) after treatment was measured using a WMicrotracker ONE in vitro biosystem (Phylumtech, Argentina) at room temperature, as described previously [26]. The motility of the untreated larvae in each comparison was used as an internal reference. Four replicates of each group were included for data analysis.

### Microscopic analysis of larval development

Worm culture mixtures were collected from each well to determine the numbers of L1s, L2s, L3s, xL3s, L4s, and sick and/or dead *H. contortus* larvae after treatment under an Olympus DP23 microscope (Olympus, Tokyo, Japan), and the length and width of these larvae were measured as described previously [17, 18, 20]. In vitro*-*cultured larvae (xL3s and L4s with developed pharynxes) were stabilised with 10% iodine and photographed under a microscope. At least 100 worms were counted and measured for larval development assessment under each condition.

### Transmission electron microscopy (TEM)

After RNAi treatment for six days in vitro, the xL3 larvae of *H. contortus* and the freshly recovered adult worms were washed in sterile physiological saline solution and fixed with 1 mL of 2.5% glutaraldehyde (diluted in 1% Triton X-100) at 4 °C overnight. The processed worms were extensively washed in phosphate buffer solution (PBS; 0.1 M, pH 7.0), fixed in 1% osmium acid for 2 h, dehydrated in gradient concentrations of ethanol solution (30, 50, 70, 80, 90, and 95%), and then embedded in low-viscosity Spurr epoxy resin (Ted Pella, CA, USA; 50% resin for 1 h, 75% resin for 3 h and pure resin overnight). The embedded sample was sliced at a thickness of 80 nm and then stained with uranyl acetate and lead citrate. The sections were observed and imaged under a Hitachi HT-7820 electron microscope (Hitachi High-Tech Co., Ltd., Shanghai, China).

### Animal experiments

The viability of RNAi-treated infective *H. contortus* larvae was assessed in Hu sheep as described previously [20]. In brief, four Hu sheep per treatment raised under helminth-free conditions after birth were infected with the infective L3s of *H. contortus* (8000 larvae per sheep) cultured in medium seeded with *E. coli* HT115 (OD = 0.3) expressing dsRNA targeting *daf-9/cyp-22a1* or *HCON_00083600* at 28 °C and 80% relative humidity for seven days, or they were infected with xL3s soaked with siRNAs targeting *bli-5* at 37 °C, 10% v/v CO_2_ and 100% relative humidity for 24 h. The weights of the sheep were recorded every day, with blood tests occasionally performed at the Center for Drug Safety Evaluation and Research (GLP) of Zhejiang University. Faecal worm egg counting was performed from 18 to 35 days post-infection, and necropsy was conducted at 35 days post-infection to assess the worm infection burden in the abomasum.

### Statistical analysis

Data from at least three biological or technical replicates were included in each assay, and the data are presented as the means ± standard error of the means (SEMs) or means ± standard deviations (SDs), respectively. Student’s t test (between two groups) or one-way ANOVA (among three or more groups) was performed using GraphPad Prism 8 (San Diego, CA, USA).* P* < 0.05 was considered to indicate statistical significance.

## Results

### daf-9/cyp-22a1 plays a crucial role in the *H. contortus* L3-to-L4 transition in vitro

**Figure 1:**
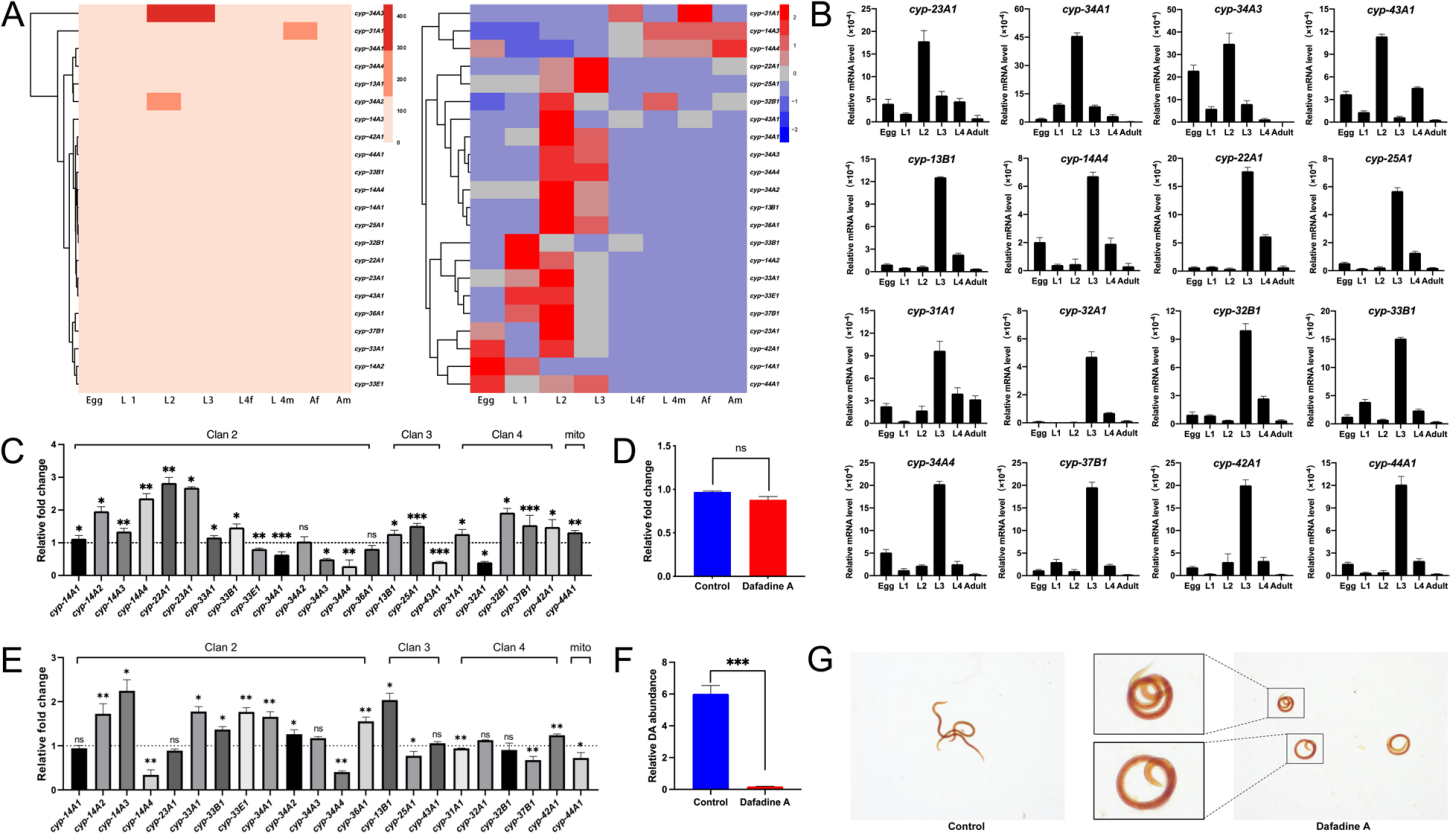
**Predominant transcription of cytochrome P450-encoding genes (*cyp*) in the infection stage of** ***Haemonchus contortus***. (**A**) Absolute and relative (Z score normalised) transcriptional heatmaps of 23 *cyp* genes among the different developmental stages of *H. contortus* (accession number: SRP026668; cf. [55]). (**B**) Relative mRNA levels of *cyp* genes to those of 18S rRNA among the different developmental stages of *H. contortus*, as determined by quantitative real-time polymerase chain reaction and 2^−ΔCT^ analyses. (**C**) Transcriptional alterations of *cyp* genes (with clan information indicated) in the exsheathed infective larvae of *H. contortus* in response to 24 h of serum exposure. (**D, E**) Changes in the transcription of *daf-9/cyp-22a1 I* and other *cyp* genes (with clan information indicated) in the exsheathed infective larvae of *H. contortus* in response to treatment with 100 μΜ dafadine A for 24 h. (**F**) Abundance of (25S)-Δ7-DA in the dafadine A-treated and -untreated *H. contortus* larvae (control). (**G**) Larval development of the exsheathed infective larvae of *H. contortus* after treatment with dafadine A compared with that of the untreated control. The error bars represent the means ± standard deviations (SDs). *, **, ***, and ns indicates *P* < 0.05, *P* < 0.01, *P* < 0.001, and not significant, respectively.

On the basis of earlier transcriptomic datasets, most *cyp* genes were highly transcribed in the L2 and L3 stages compared with the L4 and adult stages of *H. contortus* (Figure 1A; Additional file 1A; Additional file 5). Upregulation alterations of 16 *cyp* genes were detected during the transition from the xL3 stage to the L4 stage of this parasite in vitro (Additional file 1B). Transcriptional profiles of the 23 *cyp* genes among the egg, L1, L2, L3, L4, and adult stages of *H. contortus* were refined on the basis of the relative mRNA levels of these genes to those of 18S ribosomal RNA by qRT‒PCR in each stage. Dominant mRNA transcription of four *cyp* genes (i.e., *cyp-23A1*, *-34A1*, *-34A3* and *-43A1*) in the L2 stage and 12 *cyp* genes (i.e., *cyp-13B1*, *-14A4*, *-22A1*, *-25A1*, *-31A1*, *-32A1*, *-32B1*, *-33B1*, *-34A1*, *-37B1*, *-42A1* and *-44A1*) in the infective L3 stage of *H. contortus* was detected using qRT‒PCR (Figure 1B; Additional file 1C), suggesting their role in the adaptation of this parasite to parasitism.

After exposure to 10% sheep serum for 48 h in vitro, higher mRNA levels of 16 *cyp* genes (*P* < 0.05 for 12 genes) and lower levels of seven *cyp* genes (*P* < 0.05 for four genes) were detected in the xL3s of *H. contortus* than in those of the blank control (Figure 1C). The upregulated genes included family members of *cyp-13*, *-14*, *-22*, *-23*, *-25*, *-31*, *-32*, *-33*, *-37*, *-42*, and *-44*. Notably, *cyp-22a1* (also known as *daf-9*) has been reported to play a crucial role in the larval development of nematodes and exhibited the most obvious transcriptional increase in the serum-exposed xL3s of *H. contortus* (Figure 1C).

Therefore, dafadine A (a known inhibitor of *C. elegans* DAF-9) was used to inhibit the protein function of DAF-9/CYP-22A1 in the xL3s of *H. contortus*. Although treatment with dafadine A did not influence the transcription of *daf-9/cyp-22a1* (Figure 1D), it significantly (*P* < 0.05) affected the transcription of the other 16 *cyp* genes, including ten upregulated genes (i.e., *cyp-14A2*, *-14A3*, *-33A1*, *-33B1*, *-33E1*, *-34A1*, *-34A2*, *-36A1*, *-13B1* and *-42A1*) and six downregulated genes (i.e., *cyp-14A4*, *-34A4*, *-25A1*, *-31A1*, *-37B1* and *-44A1*) in xL3s (Additional file 6; Figure 1E). Treatment with dafadine A resulted in a marked and significant (*P* < 0.001) decrease in endogenous (25S)-Δ7-DA in the treated larvae (Figure 1F). Notably, the low abundance of DA and the associated alterations in other *cyp* genes led to the arrest of development with curled bodies in nearly all the treated xL3s of *H. contortus* in vitro (Figure 1G).

### Silencing of daf-9/cyp-22a1 reduces *H. contortus* infection in vivo

**Figure 2:**
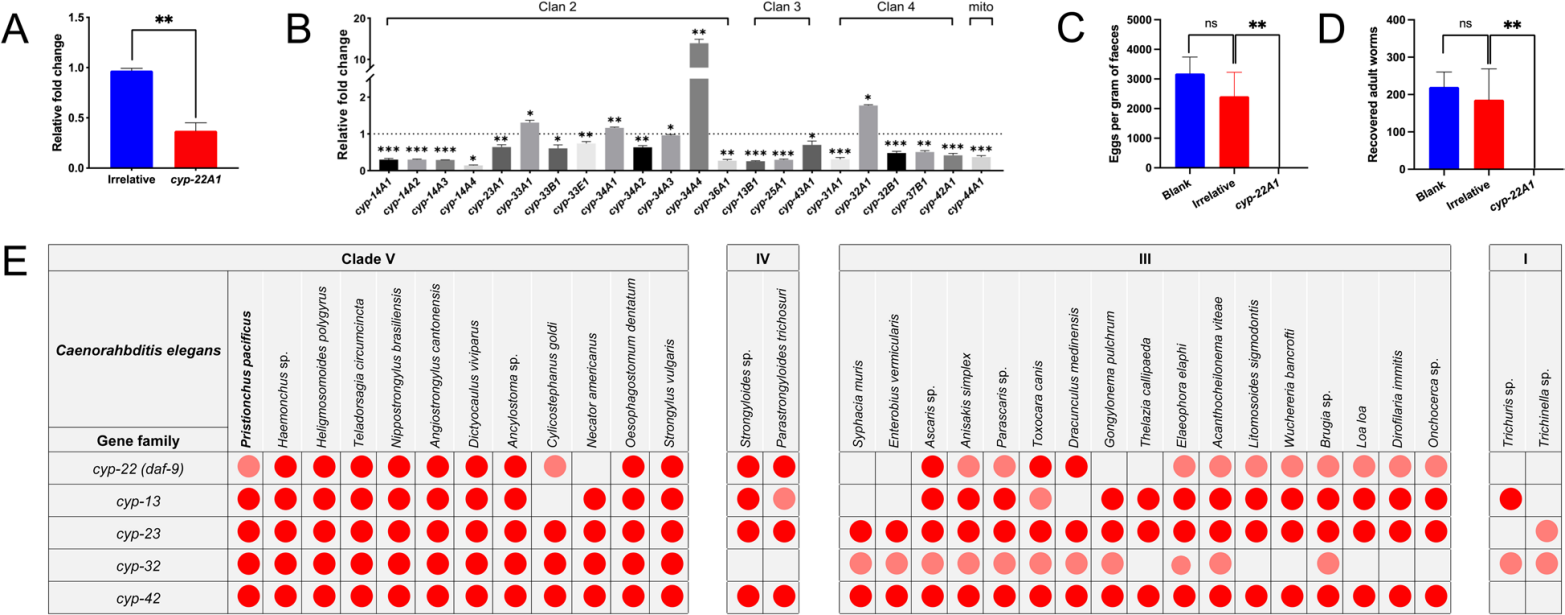
**Essentiality of** ***daf-9/cyp-22a1*** **in the establishment of** ***Haemonchus contortus*** **in vivo**. (**A**) Gene knockdown analysis of double-stranded RNA-mediated RNA interference (RNAi) of *daf-9/cyp-22a1* using a feeding method in infective *H. contortus* larvae, as determined by quantitative real-time polymerase chain reaction (qRT‒PCR) and 2^−ΔΔCT^ analyses. (**B**) Relative mRNA levels of other *cyp* genes to those of 18S rRNA among the different developmental stages of *H. contortus* treated with RNAi (*daf-9/cyp-22a1*), as determined by qRT‒PCR and 2^−ΔΔCT^ analyses. (**C**) Eggs per gram of feaces (EPG) determined for sheep infected with RNAi (*daf-9/cyp-22a1*)-treated larvae, irrelative and blank controls, at 28 days post-infection. (**D**) Number of adult worms recovered from the abomasum of Hu sheep infected with RNAi (*daf-9/cyp-22a1*)-treated larvae, irrelative and blank controls, at 35 days post-infection. (**E**) Genetic atlas of the *cyp* gene family inferred in a range of parasitic nematodes (clades I, III, IV, and V). Red dots indicate “1-to-1” orthologues, and pink dots indicate “1-to-many” or “many-to-many” orthologues.

DsRNA was designed on the basis of an experimentally verified coding sequence of *daf-9/cyp-22a1* and synthesised in vitro. Gene knockdown of *daf-9/cyp-22a1* was successfully (*P* < 0.01) achieved in the infective L3s of *H. contortus* by feeding the L1s/L2s of this parasite with bacteria expressing dsRNA (Figure 2A). RNAi (*daf-9/cyp-22a1*) also changed the transcriptions of the other 22 *cyp* genes in the infective L3s of *H. contortus*, with most of these genes significantly (*P* < 0.05) downregulated compared with those in the irrelative control (Figure 2B).

Infection experiments were conducted on sheep with the *daf-9/cyp-22a1-*silenced infective *H. contortus* larvae. A slight but not significant reduction in the number of eggs per gram (EPG) of faeces was detected in the irrelative control group compared with that in the blank control group at 28 days post-inoculation (Figure 2C). Remarkably, no eggs were detected in the faecal samples of the sheep inoculated with the silenced infective larvae at 28 days post-inoculation (Figure 2C). No adult worms were recovered from the abomasum of the sheep in the silenced groups at 35 days post-inoculation (Figure 2D).

Considering the importance of *daf-9/cyp-22a1* in the *H. contortus* L3-to-L4 transition and its conservation in nematodes of clades V, IV, and III (Figure 2E), it represents a target candidate for the control of these parasites. Apart from *daf-9/cyp-22a1*, homologues of *cyp-13*, *-23*, *-32*, and *-42* were also unequivocally identified in a range of parasitic nematodes.

### bli-5 is important in *H. contortus* L3-to-L4 moulting in vitro

**Figure 3:**
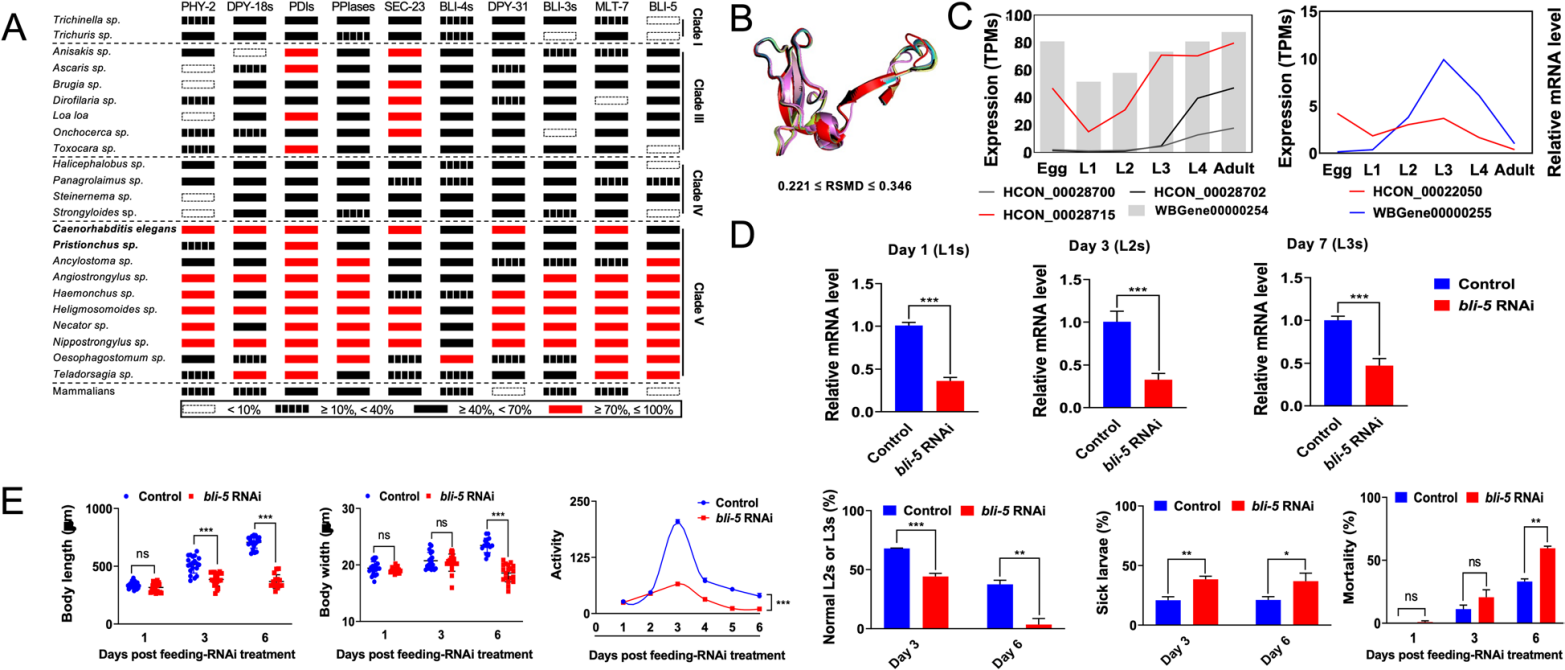
**Roles of** ***bli-5*** **in the larval moulting and development of** ***Haemonchus contortus*** **in vitro**. (**A**) Identification of gene homologues involved in collagen biogenesis in a range of nematodes (clades I, III, IV, and V). Nucleotide sequence similarities of these genes to their homologues in *Caenorhabditis elegans* were indicated on the basis of information from the WormBase ParaSite database. (**B**) Superposition of the modelled tertiary structures of BLI-5 proteins from clade V nematodes, including *Ancylostoma* sp., *Angiostrongylus* sp*.*, *Haemonchus* sp., *Heligmosomoides* sp., *Nippostrongylus* sp., *Oesophagostomum* sp., and *Teladorsagia* sp., with root-mean-square deviation (RMSD) indicated. (**C**) Comparison of the developmental transcription profiles for *bli-4* and *bli-5* orthologues between *C. elegans* (accession numbers: PRJNA13758; *bli-4*: WBGene00000254; *bli-5*: WBGene00000255) and *H. contortus* (accession number: SRP026668; cf. [55]; *bli-4*: HCON_00028700, HCON_00028702 and HCON_00028715; *bli-5*: HCON_00022050). (**D**) Gene knockdown analyses of double-stranded RNA-mediated RNA interference (RNAi) of *bli-5* using a feeding method in the infective *H. contortus* larvae after one, three and seven days of feeding, as determined by quantitative real-time polymerase chain reaction and 2^−ΔΔCT^ analyses. (**E**) Phenotypes (body length, body width, activity, normal and sick proportion, and mortality) of *H. contortus* larvae after *bli-5* silencing. Normal larvae: good motility, intact epidermis or sheath, and normal development; Sick larvae: low motility, developmental retarded or deformed, with shrinking, blistering, and swelling of the epidermis and sheath; Dead larvae: rigid bodies or partial degradation. Larvae fed bacteria containing L4440-*Bt-Cry1AC* served as the irrelative control. The error bars represent the means ± standard deviations (SDs). *, **, ***, and ns indicates *P* < 0.05, *P* < 0.01, *P* < 0.001, and not significant, respectively.

On the basis of cross-species identification, genes involved in the nematode moulting process (i.e., polypeptide synthesis, disulphide bond formation, trimerization, procollagen processing and collagen cross-linking) were also found to be conserved in nematodes and relatively conserved in mammalian hosts, particularly among the species of clade V (Figure 3A). No orthologues of the nematodes *dpy-31* and *bli-5* were predicted in their host animals (Figure 3A). Specifically, BLI-5 was more conserved in parasitic nematodes of clade V, including *H. contortus*, particularly in terms of the tertiary structures of the BPTI/Kunitz family domain (0.221 ≤ root mean square deviation ≤ 0.346) (Figure 3B). The expression patterns of *bli-4* and *bli-5* in *C. elegans* and *H. contortus* are similar (Figure 3C). As *bli-4* has already been studied in *C. elegans*, we chose *bli-5*, which showed a high transcriptional level in the L3 stage, for further investigation (Figure 3C). A high transcriptional level of the *bli-5* orthologue was also detected in the infective L3 stage of *H. contortus* (Figure 3C), suggesting that the L3-to-L4 moulting process occurred during infection establishment in host animals.

When the feeding method was used, *bli-5* was significantly (*P* < 0.001) knocked down in the free-living L1s, L2s, and infective L3s of *H. contortus* at 1, 3, and 7 days post-treatment, respectively (Figure 3D). Efficient silencing of *bli-5* resulted in shrinking (L2 and L3 stages; Additional files 3A, B, C, and G), swelling (L3 stage; Additional files 3E, F, and H) and other abnormal phenotypes (e.g., larvae trapped in the cuticle). Macrolevel effects were evident in the reduced number of normal larvae (*P* < 0.01), lower motility (*P* < 0.001), and higher sickness (*P* < 0.05) and death rates (*P* < 0.01) of the infective larvae compared to the untreated larvae (Figure 3E).

### Silencing of bli-5 affects the establishment of *H. contortus*

**Figure 4:**
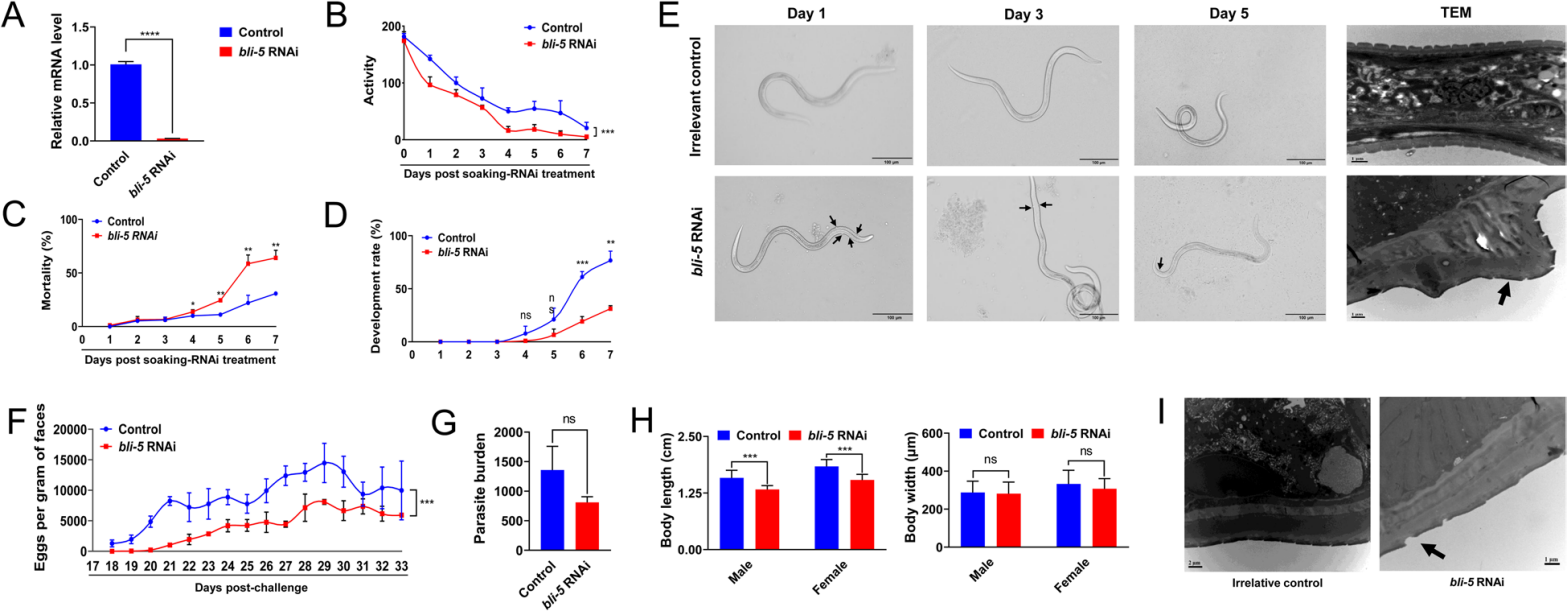
**Importance of** ***bli-5*** **in the larval moulting and development of** ***Haemonchus contortus*** **in vivo.** (**A**) Gene knockdown analysis of small interfering RNA-mediated RNA interference (RNAi) of *bli-5* using a soaking method in cultured *H. contortus* larvae after six days of incubation, as determined by quantitative real-time polymerase chain reaction and 2^−ΔΔCT^ analyses. Larval activity (**B**), mortality (**C**), and development (**D**) of the RNAi (*bli-5*)-treated larvae after one, two, three, four, five, six, and seven days of treatment. Larval activity was assessed by larval swimming motility in each well of the culture plate, which was measured by a WMicroTracker ONE (microplate format: w96u; acquisition lapse = 10 min). (**E**) Phenotype of the infective larvae of *H. contortus* observed at 1 d, 3 d, and 5 d under an optical or transmission electron microscope (TEM) following RNAi (*bli-5*) via the soaking method, with blister-like tissue indicated by black arrows. The scale bars are 1 or 100 μm. (**F**) Eggs per gram of feaces (EPG) determined for sheep infected with RNAi (*bli-5*)-treated larvae, irrelative and blank controls from 17 to 33 days post-infection. (**G**) Number of adult worms recovered from the abomasa of Hu sheep infected with RNAi (*bli-5*)-treated larvae and irrelative control at 33 days post-infection. (**H**) Body length and body width of collected adult (female and male) worms. (**I**) Transmission electron microscopy images of adult worms collected from infected sheep at 33 days post-infection. The scale bars are 1 or 2 μm. Larvae treated with siRNA targeting the *Bt-Cry1AC* gene were used as the irrelative control. The error bars indicate the means ± standard deviations (SDs), and ns indicates not significant. *** and ns indicate *P* < 0.001 and not significant, respectively.

Gene knockdown of *bli-5* was also achieved during the L3-to-L4 moulting of *H. contortus* using a soaking method in vitro (Figure 4A). Compared with the untreated control, efficient silencing of *bli-5* in the xL3s resulted in reduced activity (*P* < 0.001; Figure 4B) and an increased death rate (*P* < 0.05; Figure 4C) after 4 days of treatment and compromised the developmental transition of *H. contortus* from the L3 stage to the L4 stage at 7 days post-treatment (Figure 4D). Notably, efficient silencing of *bli-5* in these stages resulted in blister (blistering of cuticular material away from the surface) and dumpy (shortening in length) phenotypes, differing from those of the treated L3s (Figure 4E).

The xL3s of *H. contortus* soaked in medium containing siRNAs against *bli-5* for 24 h were used to infect the sheep. Compared with those in the irrelative control group, significantly lower EPG levels were detected in the faeces of sheep infected with RNAi-treated larvae from 18 to 33 days post-infection (Figure 4F). After necropsy, approximately 800 adult worms were recovered from the abomasa of sheep infected with RNAi (*bli-5*)-treated larvae, whereas 1400 adult worms were recovered from the irrelative control (Figure 4G). Although no significant effect of RNAi (*bli-5*) was detected on the body width of adult worms, a decreased body length (dumpy-like phenotype) was detected for the adult female (*P* < 0.001) and male worms (*P* < 0.001) recovered from the sheep infected with treated larvae compared with that of the irrelative control group (Figure 4H). Neither a blister phenotype nor obvious differences were identified in the adult worms recovered from the sheep that were infected with RNAi (*bli-5*)-treated larvae (Figure 4I). The efficacy of siRNA-mediated gene knockdown was measured in the recovered adult worms. Compared with the irrelative control, slightly but not significantly lower transcription of *bli-5* was detected in the adult worms (Additional file 2).

### Silencing of haem utilisation-associated HCON_00083600 compromises the parasitism of *H. contortus* in vivo

**Figure 5:**
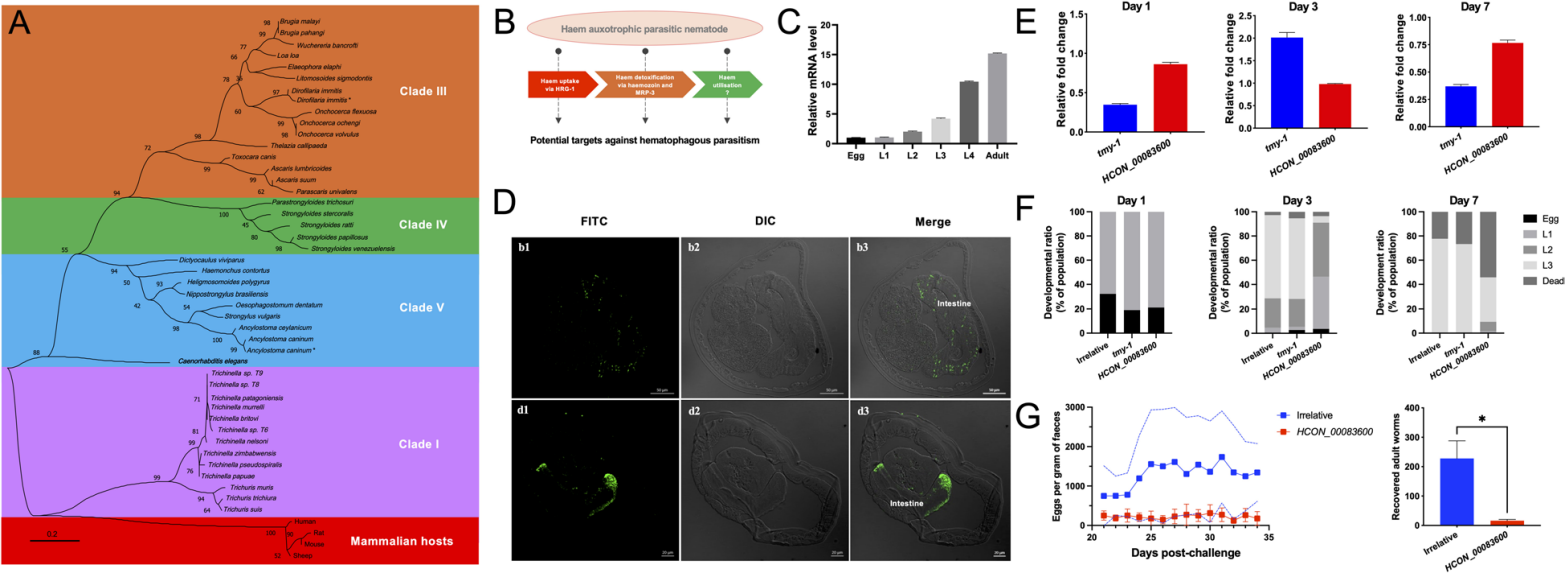
**Importance of the haem utilisation gene** ***HCON_00083600*** **in** ***Haemonchus contortus*** **in vitro and in vivo.** (**A**) A maximum likelihood tree of the HRG-1 amino acid sequences was constructed for a range of parasitic nematodes (clades I, III, IV and V) and mammalian hosts, such as sheep, mice, rats and humans. (**B**) Schematic demonstrating haem uptake by the unique haem transporter HRG-1, haem detoxification by haemozoin formation and MRP-3 efflux, and haem utilisation in the haem auxotrophic, blood-feeding nematode *H. contortus*. (**C**) Relative mRNA levels of *HCON_00083600* to those of 18S rRNA among the different developmental stages of *H. contortus*, as determined by quantitative real-time polymerase chain reaction and 2^−ΔCT^ analyses. (**D**) Tissue expression of HCON_00083600 protein in adult *H. contortus* females and males, as determined using a polyclonal antibody-based indirect immunofluorescence assay. (**E**) Gene knockdown analysis of small interfering RNA-mediated RNA interference (RNAi) of *HCON_00083600* using a soaking method in the cultured larvae of *H. contortus* after one, three and seven days of incubation, as determined by quantitative real-time polymerase chain reaction and 2^−ΔΔCT^ analyses. Larvae treated with siRNAs targeting the *tmy-1* and *Bt-Cry1AC* genes were used as positive and irrelative controls, respectively. (**F**) Larval development of RNAi (*HCON_00083600*)-treated larvae after one, three and seven days of incubation, in terms of the ratios of unhatched eggs and the first- (L1s), second- (L2s), and third-stage (L3s) larvae of *H. contortus* in vitro. (**G**) Eggs per gram of faeces (EPG) determined for sheep infected with RNAi (*HCON_00083600*)-treated infective larvae, compared with the irrelative control, from 20 to 34 days post-infection. Number of adult worms recovered from the abomasa of Hu sheep infected with RNAi (*HCON_00083600*)-treated larvae and irrelative control at 35 days post-infection. The error bars indicate the means ± standard deviations (SDs), and ns indicates not significant. **** and * indicate *P* < 0.0001 and *P* < 0.05, respectively.

The haem transporter HRG-1 is encoded by a unique gene in parasitic nematodes that plays vital roles in haem uptake and utilisation and thus represents a target candidate for the control of these pathogens, particularly blood-feeding species such as *H. contortus*. This gene is conserved in nematodes of clades V, IV, III and I, as well as in mammalian hosts, including sheep, mice, rats and humans (Figure 5A), limiting its potential to treat nematode infection in animals. A novel gene, *HCON_00083600,* was predicted to be involved in HRG-1-associated haem utilisation (Figure 5B), with no homologue inferred in mammalian hosts, representing a preferable target in the control of barber’s pole worm.

On the basis of the relative mRNA level of *HCON_00083600* to that of *actin-1*, high transcriptional levels of this gene were detected in the blood-feeding L4 and adult stages of *H. contortus* (Figure 5C). Using polyclonal antibodies against recombinant HCON_00083600 protein, an indirect immunofluorescence assay was conducted on sections from female and male adults. HCON_00083600 protein was expressed predominantly in the intestine of blood-feeding *H. contortus* worms, with a punctate distribution (Figure 5D).

A feeding method was used to mediate gene knockdown of *HCON_00083600* in the free-living (L1, L2, and L3) stages of *H. contortus*. A slightly lower transcriptional level of *HCON_00083600* was detected in the treated larvae of this parasite after three days but not after 7 days (Figure 5E), resulting in compromised larval development and a higher death rate of the infective larvae in vitro than in the irrelative control (Figure 5F). The infective larvae that survived the RNAi (*HCON_00083600*) were used to inoculate the sheep. In these sheep, compared with the irrelative control, neither eggs nor adult worms were detected in the faeces from 21 to 63 days post-inoculation (Figure 5G).

## Discussion

Using an integrative approach, three major genes (*cyp-22a1/daf-9*, *bli-5*, and *HCON_00083600*) involved in larval activation, moulting and haem utilisation were identified in the blood-feeding nematode *H. contortus*. dsRNA- and/or siRNA-mediated gene knockdown of these genes were used for functional validation in vitro and for the application assessment of RNAi technologies in *H. contortus* infection intervention in vivo.

The establishment of infection involves a series of steps in which the infective larvae of *H. contortus* enter the host abomasum, shed their protective sheath, and begin development, namely, the activation of infective larvae. Larval activation has been extensively studied at the molecular level, revealing major molecules involved in this key biological process of nematode infection. For instance, the homologues of *daf-9/cyp-22a1*, a key regulator of *C. elegans* larval development and adult longevity [56, 57], have been consistently reported to play a role in the activation of infective larvae of parasitic nematodes, including *Strongyloides stercoralis* (the human threadworm; [58, 59]), *H. contortus* (the barber’s pole worm; [17]), and *Nippostrongylus brasiliensis* [60], on the basis of in vitro assays. In the current study, host serum was further used to stimulate artificially exsheathed infective *H. contortus* larvae to preliminarily simulate the early blood-feeding stage of this parasite and to identify more genes involved in this process. Interestingly, *daf-9* was found to be the most strongly expressed *cyp* gene in the stimulated larvae, and chemical inhibition of the DAF-9 protein compromised subsequent larval development (cf. [51]). Although the crucial role of *daf-9* in larval development after activation was not surprising, because DAF-9 is required for the biosynthesis of dafachronic acids (DAs), which act as ligands for the nuclear hormone receptor NHR/DAF-12 (it determines dauer entry or exit in the free-living nematode *C. elegans* [57, 61–63]), *daf-9/cyp-22a1* represents a potential target in the adaptation to parasitism by *H. contortus* larvae in host animals.

The resulting activation of infective larvae is essential for the L3–L4 transition of *H. contortus*. Growing evidence indicates that relatively conserved mechanisms regulate developmental transition processes in invertebrates and vertebrates, including moulting in nematodes [64, 65]. The key molecules involved in the collagen biogenesis cascade are highly conserved among nematode species, with highly similar orthologues also inferred in host animals. For example, PDIs, PPIases and SEC-23, which are associated with disulphide bond formation, trimerization and secretion from the endoplasmic reticulum (ER) to the cytoplasm, respectively, shared more than 40% of the amino acid sequence between nematodes and their hosts. Among the analysed molecules, only *dpy-31* and newly identified *bli-5* are absent in host animals. The function and inhibition of *dpy-31* in parasitic and free-living nematodes have been well described elsewhere [66–68], and knockdown of *bli-5* resulted in a blister phenotype at the L4 and adult stages of *C. elegans* [68, 69]. However, the role of *bli-5* in parasitic nematodes is unclear. Here, it was found that *bli-5* is required for free-living L1 and L2, particularly infective L3 to L4 moulting of *H. contortus*. RNAi (*Hc-bli-5*) was linked to blister-like spots in the larval stages of *H. contortus* in vitro, particularly during the moulting processes from L3 to L4 of this parasite, representing a potential target in larval development within host animals.

In our recent studies, the unique haem transporter-encoding gene *hrg-1* and the ABC transporter-encoding gene *mrp-3* were reported to be promising target candidates for the control of *H. contortus* in sheep [19, 20]. However, homologues of *hrg-1* and *mrp-3* are well conserved in a range of host animals, including sheep, goats, mice, rats and humans, hindering the application of these targets in the intervention of blood-feeding nematodes. A gene that is required for haem utilisation in nematodes but absent from host animals is warranted and should preferably be identified on the basis of salient information on the haem biology of nematodes [70–73]. HCON_00083600, a protein of unknown function, appeared in the HRG-1 pull-down assay and is not predicted to be an alternative target in sheep or other mammals. The highest mRNA transcription and dominant protein distribution were detected in the intestine of adult worms and in the gonads of male worms, which is consistent with the role of HRGs in *H. contortus*, suggesting roles in the haem biology of this blood-feeding nematode. HRG-1 functions at the intestinal membrane (haem uptake) and the endolysosomal system (haem transport) [20, 72, 74], and HCON_00083600 is not a predicted transmembrane protein; thus, it is more likely to play a role in haem utilisation in the endolysosomal system. Importantly, RNAi (HCON_00083600) resulted in compromised larval development and increased the death of infective *H. contortus* larvae. These results indicate the importance of HCON_00083600 in *H. contortus* and its potential as an intervention target, although more investigations are warranted to further elucidate the role of this gene.

The potential targets of *H. contortus* that have been identified in in vitro assays should be preferably tested in vivo, particularly in terms of nematode infection and establishment in host animals. In the current study, the importance of *daf-9/cyp-22a1*, *bli-5* and *HCON_00083600* in the adaptation to *H. contortus* parasitism was tested in sheep using RNAi-treated infective larvae of this parasite. Compared with the control, gene silencing of *daf-9/cyp-22a1*, *bli-5* or *HCON_00083600* in the infective larvae of *H. contortus* resulted in reduced egg production and a smaller number of established adult worms in host animals. Although it is still not clear whether the RNAi-treated larvae establish infection and develop to the reproductive stage (*bli-5* was not efficiently knocked down after 33 days post-infection), the importance of *daf-9/cyp-22a1*, *bli-5* and *HCON_00083600* in nematode infection and their potential as target candidates in the control of *H. contortus* have been unequivocally validated in host animals. Since the United States Food and Drug Administration (FDA) and European Commission (EC) approval of ONPATTRO (Patisiran), the first RNAi therapeutic for the polyneuropathy of hereditary transthyretin-mediated (hATTR) amyloidosis in adults [75], RNAi has been proposed as a clinical treatment strategy and as a functional genomic tool. This technique holds promise for the intervention of nematode infections in domestic animals, particularly at the early stage of infection, although challenges remain in effective siRNA or dsRNA delivery.

In addition to the exciting findings, there are also limitations in understanding the roles of the target candidates in nematode infection and in advancing the application of RNAi technologies in clinical trials. (1) Given the roles of *cyp* genes in the multifunctional oxidase system of life forms [76], clearly, the marked contraction of the *cyp* gene family in parasitic nematodes strongly indicates their essentialities. However, considering the broad effects (possibly toxic effects; [51]) on the *H. contortus cyp* gene family in vitro, whether RNAi or dafadine A can be used as an anthelmintic synergist by compromising larval development in host animals remains unclear. (2) Although both dsRNA-mediated RNAi by a feeding method and siRNA-mediated RNAi by a soaking method achieved sufficient gene knockdown in *H. contortus* and effective intervention of nematode infection in sheep, these methods were pre-conducted on the infective larvae of this parasite. Unlimited delivery of RNAi agents to any stage of nematode infection is still warranted. Lentivirus-mediated RNAi might be one approach for overcoming this issue, which remains to be modified for parasitic worms [77, 78], particularly for clinical trials in the future.

In conclusion, *daf-9/cyp-22a1*, *bli-5*, and *HCON_00083600* are essential genes involved in larval development after the activation, moulting and blood-feeding of *H. contortus*, representing intervention target candidates for the adaptation to parasitism by this parasitic nematode in host animals. A better understanding of nematode biology and progress in the modification of RNAi technologies are warranted for improved control of *H. contortus* and related parasitic worms.

## Supplementary Information

**Additional file 1: Nomenclature of** ***Haemonchus contortus*** **cytochrome P450 (CYP)-encoding genes and their transcriptional profiles among different developmental stages**. (**A**) A maximum likelihood phylogenetic tree based on the amino acid sequences of 23 *H. contortus* CYPs, 80 *Caenorhabditis elegans* CYPs, and the outgroup CYP6A2 from Drosophila melanogaster. Bootstrap values after 1000 replications are shown in bubbles at each clade. (**B**) Absolute and relative (Z score-normalised) transcriptional heatmaps of 23 *cyp* genes in third-stage larvae (L3s), exsheathed L3s (xL3s), and in vitro-cultured fourth-stage larvae (L4s) of *H. contortus* (accession number: SRP136037; cf. [47]). (**C**) Relative mRNA levels of selected *cyp* genes to those of 18S rRNA among the different developmental stages of *H. contortus*, as determined by quantitative real-time polymerase chain reaction and 2^−ΔCT^ analyses.

**Additional file 2: Gene knockdown analysis of** ***bli-5*** **RNA interference (RNAi) in adult worms of** ***Haemonchus contortus*** **in vivo**. The infective H*. contortus* larvae were soaked in small interfering RNA in vitro for 24 h and then used to infect the sheep. Adult worms were collected from the abomasa of infected sheep at 35 days post-infection. The error bars indicate the means ± standard deviations (SDs), and ns indicates not significant.

**Additional file 3: Moulting phenotypes of** ***Haemonchus contortus*** **larvae during development after** ***bli-5*** **silencing via the feeding method.** (**A**–**C**) Moulting defects observed on day 3 post-RNAi, with black arrows indicating “tight-suit” phenotypes and the old cuticle being ensheathed. (**D**) Development of the irrelative control larvae fed bacteria containing L4440-*Bt-Cry1AC*. (**E**–**H**) Shrinkage (red arrows), swelling and incomplete moulting (black arrows) of treated larvae after treatment for six days (I) compared with the irrelative control.

**Additional file 4: Primer sets used in this study**.

**Additional file 5: Curated nomenclature of** ***cyp*** **genes in** ***Haemonchus contortus.***

**Additional file 6: Integrated prediction of cytochrome P450s in major parasitic nematodes.**

## Data Availability

All the data generated or analysed during this study are included in this published article and its supplementary information files. *Cyp* sequences of *Haemonchus contortus* were uploaded to NCBI GenBank (Accession number: BankIt2908146, [52]).
