# Supplementary figures and images for: Experimental validation of RNA interference technologies for improved control of barber’s pole worm
Source: Vet Res. 2025 Oct 14;56:194. doi: 10.1186/s13567-025-01633-6 (PMC12522317; doi:10.1186/s13567-025-01633-6)

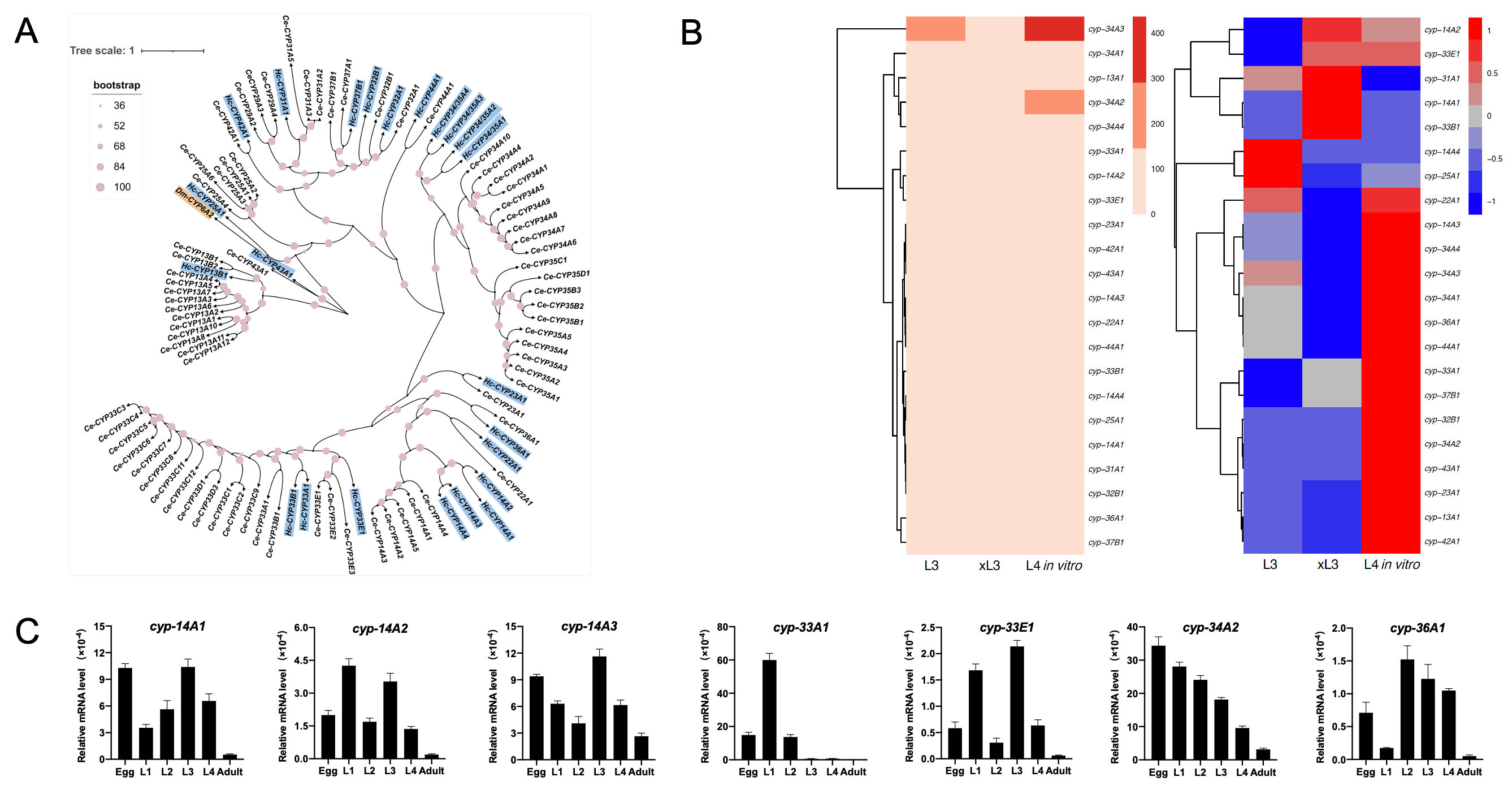

Supplement: Supplementary file 1 — Additional file 1: Nomenclature of Haemonchus contortus cytochrome P450 (CYP)-encoding genes and their transcriptional profiles among different developmental stages. (A) A maximum likelihood phylogenetic tree based on the amino acid sequences of 23 H. contortus CYPs, 80 Caenorhabditis elegans CYPs, and the outgroup CYP6A2 from Drosophila melanogaster. Bootstrap values after 1000 replications are shown in bubbles at each clade. (B) Absolute and relative (Z score-normalised) transcriptional heatmaps of 23 cyp genes in third-stage larvae (L3s), exsheathed L3s (xL3s), and in vitro-cultured fourth-stage larvae (L4s) of H. contortus (accession number: SRP136037; cf. [47]). (C) Relative mRNA levels of selected cyp genes to those of 18S rRNA among the different developmental stages of H. contortus, as determined by quantitative real-time polymerase chain reaction and 2−ΔCT analyses. [file 13567_2025_1633_MOESM1_ESM.tiff]

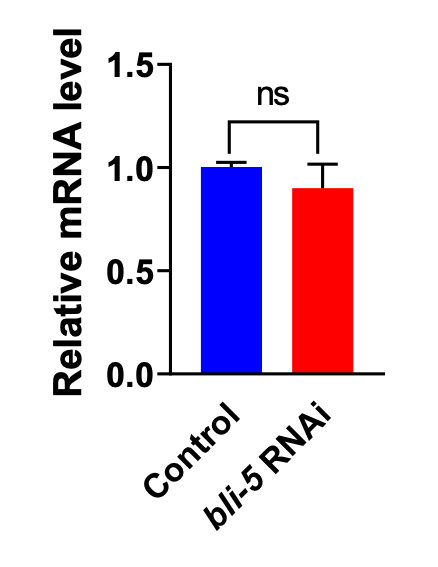

Supplement: Supplementary file 2 — Additional file 2: Gene knockdown analysis of bli-5 RNA interference (RNAi) in adult worms of Haemonchus contortus in vivo. The infective H. contortus larvae were soaked in small interfering RNA in vitro for 24 h and then used to infect the sheep. Adult worms were collected from the abomasa of infected sheep at 35 days post-infection. The error bars indicate the means ± standard deviations (SDs), and ns indicates not significant. [file 13567_2025_1633_MOESM2_ESM.tiff]

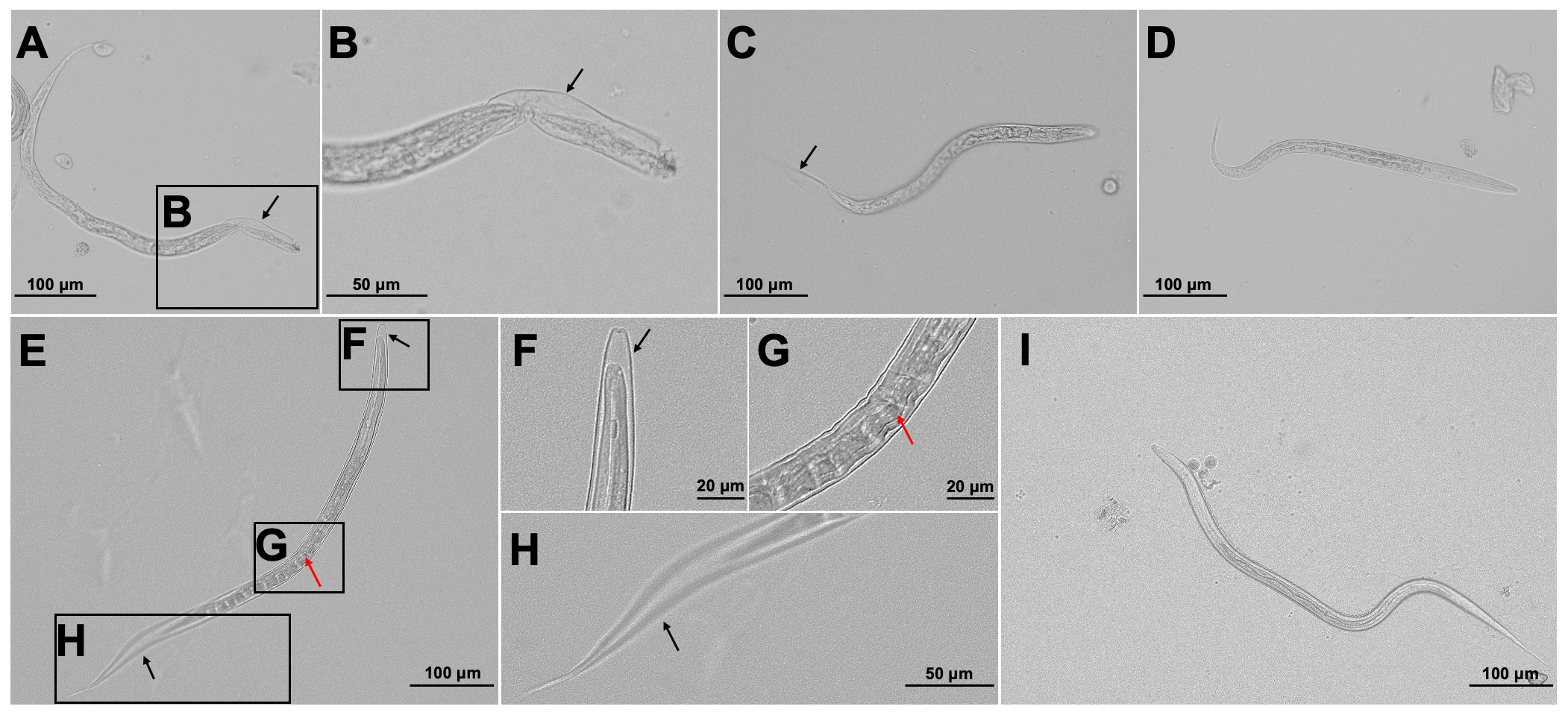

Supplement: Supplementary file 3 — Additional file 3: Moulting phenotypes of Haemonchus contortus larvae during development after bli-5 silencing via the feeding method. (A–C) Moulting defects observed on day 3 post-RNAi, with black arrows indicating “tight-suit” phenotypes and the old cuticle being ensheathed. (D) Development of the irrelative control larvae fed bacteria containing L4440-Bt-Cry1AC. (E–H) Shrinkage (red arrows), swelling and incomplete moulting (black arrows) of treated larvae after treatment for six days (I) compared with the irrelative control. [file 13567_2025_1633_MOESM3_ESM.tiff]
